# Systematic design of auxotrophic strains and media conditions to probe metabolic functions in *E. coli*

**DOI:** 10.1371/journal.pcbi.1014469

**Published:** 2026-06-29

**Authors:** Roghaye Mohammadbeygi, Patrick F. Suthers, Fang-Yu Chung, Brian F. Pfleger, Costas D. Maranas

**Affiliations:** 1 Department of Chemical Engineering, The Pennsylvania State University, University Park, Pennsylvania, United States of America; 2 Department of Chemical and Biological Engineering, University of Wisconsin–Madison, Madison, Wisconsin, United States of America; Pacific Northwest National Laboratory, UNITED STATES OF AMERICA

## Abstract

Despite progress in automated gene annotation, many deficiencies and knowledge gaps remain, even for well-studied organisms. Of particular concern is the accuracy and detail of annotations for transporters of various organic substrates and products of metabolism and for enzymes that do not share sequence homology with well-characterized strains. Unfortunately, annotation errors present in earlier genome-scale metabolic (GSM) models propagate to newer models with few opportunities for later correction. Here, we introduce a systematic computational procedure that applies the *Escherichia coli* genome-scale metabolic model *i*ML1515, extended with transcriptional regulatory rules, to design auxotrophs that can grow on glucose but fail to grow on different carbon substrate(s) unless rescued with the addition of an ORF encoding a complementation metabolic function (transport and enzymatic reactions). Using the *E. coli* GSM model supplemented with regulatory rules that quantify growth/no growth outcomes on different organic substrates, we identified 258 distinct auxotrophic designs (97 single-gene, 142 double-gene, and 19 triple-gene knockouts) for which specific single functions can uniquely complement them. Experimental validation of 61 single-knockout strains demonstrated 59% confirmed auxotrophy and 28% partial auxotrophy. We envision that this collection of auxotrophic strains can be used to disambiguate the metabolic role of unannotated or poorly annotated genes.

## Author summary

Understanding gene function remains one of the central challenges in microbiology. Roughly 35% of *E. coli* genes have no confirmed metabolic role, and annotation errors in earlier genome-scale models propagate forward with few opportunities for correction. We developed and applied a computational framework that systematically designs conditional auxotrophic *E. coli* strains that cannot grow on specific alternative carbon sources unless a particular metabolic function is restored. By predicting which gene deletions create these growth defects and which complementing functions repair them, the framework builds a library of diagnostic growth conditions whereby introducing an unknown gene and observing whether growth recovers can reveal what that gene does metabolically. Using the *i*ML1515 genome-scale metabolic model together with 1,643 Boolean regulatory rules, we identified 258 unique auxotrophic strain designs spanning single, double, and triple gene knockouts and covering 220 distinct metabolic functions (56 transport, 164 enzymatic). Experimental validation of 61 single-knockout strains confirmed auxotrophy in 59% of cases and revealed partial auxotrophy in 28% of cases. Triple knockout designs uncovered a deeper tier of metabolic redundancy, including three DAHP synthase isozymes that must all be removed before aromatic amino acid biosynthesis fails. The complete design library and computational pipeline are available at https://github.com/maranasgroup/auxotroph-design.

## 1. Introduction

Microbial growth or the absence thereof upon gene deletion(s) is, along with alternate carbon substrate utilization, a cornerstone test used in various contexts in biotechnology and fundamental biology [1]. The answer to this *in vivo* question (i.e., growth/no growth) provides important information regarding the presence of alternate metabolic pathways, transporter systems and specific details of the organism’s metabolism [2]. Gene essentiality studies, particularly those utilizing the Keio collection of *Escherichia coli* single-gene knockouts [3], are crucial for identifying genes indispensable for survival under specific conditions. This understanding not only complements growth/no-growth experiments for single deletion mutants and alternate carbon substrates in evaluating genome-scale metabolic (GSM) models but also leads to the exploration of auxotrophies, where organisms require external sources of certain essential compounds resulting from genetic deficiencies.

Auxotrophy is defined as the inability of an organism to synthesize a particular organic compound required for its growth under specific conditions, thus requiring external supplementation for survival [4]. In microbial genetics, conditional auxotrophic mutants have been extensively used to elucidate the function(s) of genes involved in metabolic pathways and nutrient synthesis [5]. Moreover, designing defined media tailored for non-model organisms is critical to support their growth and study their unique metabolic pathways, especially when working with auxotrophic mutants. Importantly, conditional auxotrophs can grow without supplementation under permissive conditions but require the compound under restrictive conditions. The concept has evolved to include conditional auxotrophy, where organisms require supplementation only under specific growth conditions while maintaining prototrophy under permissive conditions. Such designs have proven valuable across diverse applications: engineered *E. coli* strains made NADPH-auxotrophic by deleting all NADPH-producing pathways except for one serve as biosensors for studying NADPH regeneration mechanisms [6]; prototrophic deletion mutant collections in yeast facilitate metabolomics and systems biology studies [7]; and histidine auxotrophic mutants in S*accharomyces* cerevisiae enable identification of genes associated with histidine biosynthesis through complementation analysis [8,9]. Furthermore, auxotrophic markers such as URA3, LEU2, and TRP1 remain essential tools for confirming gene disruptions in yeast and other fungi [10,11], while integration of the phosphoketolase shunt into auxotrophic *E. coli* strains demonstrates how metabolic rewiring can be systematically studied [12].

A key concept underlying this framework is conditional gene essentiality, where gene or genes may be dispensable for growth under one condition (e.g., glucose) yet become essential under another (e.g., an alternative carbon source) when the metabolic function it encodes is required. Genome-scale metabolic (GSM) model predictions benchmarked against the Keio collection achieve over 90% accuracy on growth/no-growth outcomes across diverse substrates, with disagreements clustering around transporter deletions and peripheral metabolic genes where in vivo redundancy is underrepresented in the model [2,13]. These discrepancies arise from metabolic redundancy where alternative pathways or isozymes that compensate for a gene deletion are present in vivo but are absent or incomplete in the model. Moreover, regulatory effects that alter pathway availability in a substrate-specific manner also significantly contribute. Exploiting conditional essentiality as a diagnostic tool, rather than viewing discrepancies as model failures, provides the foundation for the present framework. Extending this logic to pairs and triples set of gene deletions introduces synthetic lethalities: conditions where neither deletion alone eliminates growth, but their combination does, because each gene contributes to a non-redundant portion of a shared metabolic function [14]. Synthetic lethal pairs are particularly valuable for probing biosynthetic pathways where single knockouts are lethal under all conditions, including glucose, and therefore cannot be detected by the conditional essentiality screen alone.

**Fig 1:**
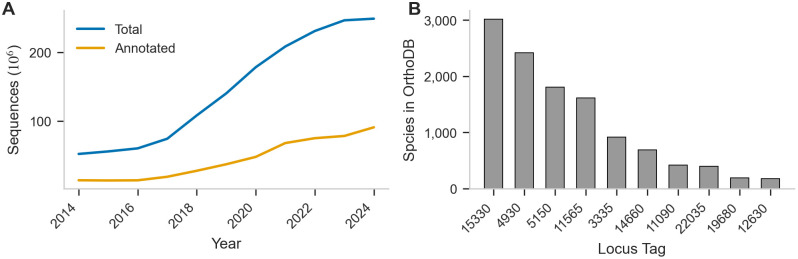
Sequence-annotation gap and conservation of uncharacterized genes (A) Growth in Total vs. Annotated Sequences from 2014-2024. The graph shows the number of sequences in millions (10⁶), with the blue line representing total sequences and the orange line showing annotated sequences. The data demonstrates an increasing gap between the total number of sequences and those with annotations over the ten-year period [15], **(B)** Distribution of the ten most prevalent *E. coli* genes of unknown function (y-genes) across microbial species. The bars show the number of species in OrthoDB containing homologs for each E. coli locus tag. These highly conserved y-genes appear in over 12,000 different microorganisms, suggesting potential functional importance despite their uncharacterized status.

Despite extensive applications of auxotrophy in functional genomics, significant gaps remain in the understanding of gene function even in well-studied organisms (Fig 1 A). Of particular concern is that 35% of open reading frames (ORFs) in *E. coli* [16,17] and 18% of ORFs in *Bacillus subtilis* [18–21] remain functionally uncharacterized. This annotation crisis is amplified through homology-based propagation: OrthoDB [22] indicates that assigning roles to ten *E. coli* genes of unknown function would improve thousands of annotations across other species (Fig 1 B). Current automated annotation tools, including BLAST [23], SEED [24], and DREAM [25], rely primarily on sequence similarity for functional assignment. However, these tools are far from perfect, as relatively low sequence similarity can lead to generic or incorrect inferences. For example, gene *selO* in *E. coli* was initially annotated as inactive or having an unclear function; only later experimental evidence [26] revealed its AMP transfer function. Such annotation errors present in earlier genome-scale metabolic models propagate to newer models with few opportunities for correction, underscoring the critical need for experimental validation approaches.

Computational efforts to address gene function discovery have increasingly employed constraint-based modeling approaches. Flux Balance Analysis (FBA) [27] on reconstructed metabolic networks enables prediction of gene deletion impacts on cellular metabolism [28,29]. Previous computational methods for auxotroph identification include Auxofind [5], which predicted auxotrophies in over 1,300 Gram-negative bacterial strains but identified only 54 auxotrophic strains, highlighting species-dependent metabolic differences. The use of bilevel optimization has been leveraged for auxotroph design. OptAux [30] designs auxotrophic dependencies by maximizing obligate uptake of a target metabolite, engineering strains for syntrophic co-cultures or biocontainment [31,32], rather than for systematic gene function discovery. A framework that couples carbon substrate-specific conditional auxotrophy with regulatory constraints and rescue function enumeration is therefore needed to convert auxotrophic phenotypes into selective conditions for experimental functional assignment of uncharacterized ORFs.

The evolution of genome-scale metabolic models has provided increasingly sophisticated platforms for computational design. The *E. coli* MG1655 model *i*ML1515, comprised of 1,516 genes, 2,712 reactions, and 1,877 unique metabolites, represents the most comprehensive *E. coli* metabolic reconstruction and updated gene-protein-reaction associations [33]. *i*ML1515 has been extensively benchmarked against the Keio collection growth phenotype data and achieves high accuracy in predicting gene essentiality across diverse substrate conditions. Integration of transcriptional regulatory information with metabolic models further improves phenotype prediction accuracy by capturing the interplay between gene regulation and metabolic state under different carbon substrates [31]. Nevertheless, gaps between model predictions and experimental results persist, particularly for genes encoding transporters of organic substrates and enzymes lacking sequence homology with well-characterized proteins.

Recent technological advances have enhanced capabilities for experimental validation of computational predictions. RNA-guided Cas9 nuclease technology has transformed the creation of multiple auxotrophic markers in industrial strains, facilitating systematic genetic manipulations [32,34]. High-throughput screening platforms enable rapid phenotyping across multiple growth conditions, while microbial biosensors leveraging substrate auxotrophy couple target chemical synthesis with easily detectable proxy metabolites [35–37]. These biosensors demonstrate how auxotrophy-based designs can be applied to studying gene function through growth-coupled selection. The convergence of computational design capabilities with experimental validation technologies creates opportunities for systematic design-build-test cycles in functional genomics.

Despite these advances, a systematic framework for designing auxotrophic strains across different organisms and broader applications remains lacking. Many existing methods are tailored to specific research questions or organisms, limiting their adaptability for comprehensive functional studies. The challenge is particularly acute for transporters and enzymes involved in alternative carbon metabolism, where sequence-based annotation frequently fails. Moreover, designing strains that exhibit conditional auxotrophy requires careful integration of regulatory information with metabolic network structure. These challenges highlight the need for a framework that systematically covers the condition space (*i.e.*, 46 aerobic carbon sources), the gene space (*i.e.*, all 1,516 *i*ML1515 genes), and the rescue function space. The experimental space of uncharacterized ORFs can be made tractable through three prioritization strategies applied prior to complementation screening: condition-specific transcriptomic profiling to identify ORFs induced on the auxotrophic substrate, genomic co-localization with characterized pathway operons, and phylogenetic profiling to identify ORFs that co-occur with known pathway genes across species. Together these filters reduce the candidate space from thousands of uncharacterized ORFs to tens or hundreds per auxotrophic design, making library-scale complementation screening feasible.

Here, a systematic computational procedure is introduced that applies genome-scale metabolic models of *Escherichia coli* to design conditional auxotrophic strains for functional gene discovery. The approach generates strains that grow normally on glucose but fail to grow on alternative carbon substrates unless rescued with specific genetic complementation. The contribution is the systematic design of these diagnostic phenotypes, where each auxotrophic strain serves as a selective platform for downstream complementation: introducing an unknown gene and observing whether growth is restored directly reveals its metabolic function. Using the *E. coli* GSM model *i*ML1515 supplemented with extended substrate-specific regulatory rules, 258 distinct auxotrophic designs were computationally identified, each requiring specific functions for growth restoration. These designs encompass 56 transport functions and 164 enzymatic functions, providing comprehensive coverage of metabolic capabilities. Experimental validation of 61 designed strains demonstrated 59% confirmed auxotrophy and 28% partial auxotrophy, establishing the utility of this approach for systematic functional genomics. This collection of auxotrophic strains and the underlying computational framework provide resources for elucidating functions of uncharacterized ORFs, with potential applications extending to other organisms and biotechnological objectives. Computational pipeline can be found at: https://github.com/maranasgroup/auxotroph-design

## 2. Methods

**Fig 2:**
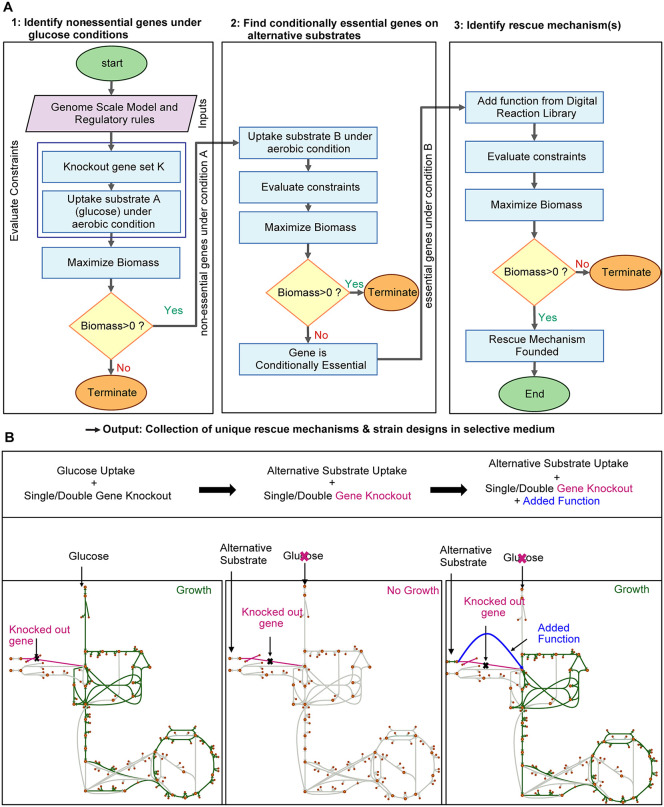
Computational workflow for gene function characterization through designed selective growth conditions (A) Computational workflow for designing selective growth conditions to elucidate gene functions. The workflow comprises three sequential steps: identification of non-essential genes under glucose conditions, evaluation of conditional essentiality under alternative substrates, and identification of rescue mechanisms using a Digital Reaction Library. The process outputs unique rescue mechanisms and strain designs for selective media, enabling functional characterization of uncharacterized genes. Decision points evaluate growth viability through biomass production (Biomass>0), **(B)** Schematic representation of the workflow illustrating how designed auxotrophic strains are used in growth/no-growth tests to determine gene function. Green reactions indicate flux-carrying routes, gray represent routes with zero flux, and the red denotes the affected reaction(s) resulting from the gene knockout.

A systematic computational framework was developed to identify strain designs that can elucidate functions of uncharacterized open reading frames (ORFs) through their growth requirements. The workflow, illustrated in Fig 2 A, consists of three sequential computational stages that integrate genome-scale metabolic modeling with regulatory constraints: (1) identification of non-essential genes under glucose conditions through systematic single-gene and paired-gene deletions with biomass optimization, (2) evaluation of these genes under alternative substrates to identify conditionally essential genes, and (3) identification of potential rescue mechanisms using functions from a digital reaction library. Each stage employs metabolic modeling to predict phenotypes, with growth viability assessed through biomass production calculations (Biomass>0) at key decision points, enabling the rational design of strains where growth depends on specific metabolic functions (Fig 2 B). This systematic approach creates a platform for functional genomics applications by establishing clear phenotype-to-function relationships between rescue functions and uncharacterized gene roles.

### 2.1. Genome-Scale metabolic model selection and computational analysis

The *E. coli* MG1655 genome-scale metabolic model *i*ML1515 [33], comprising 1,516 genes, 2,712 reactions and 1,877 unique metabolites, served as the basis for computational analyses. Flux Balance Analysis (FBA) simulations were performed using COBRApy [38] framework to predict metabolic phenotypes under different genetic and environmental conditions. A non-growth associated ATP maintenance of 8.39 mmol gDW^-1^h^-1^, carbon substrate uptake of 10 mmol gDW^-1^h^-1^ and oxygen uptake of 20 mmol gDW^-1^h^-1^ were used throughout [29].

### 2.2. Regulatory rules evaluation

Transcriptional regulatory constraints were incorporated using regulatory flux balance analysis (rFBA) [28]. The *i*MC1010v2 Boolean rule set [31] provides regulatory rules for 1,006 genes; coverage was extended to the 637 genes present in *i*ML1515 but absent from *i*MC1010v2 by constructing new rules from RegulonDB v12 S/C-confidence TF-gene interactions [39][40], yielding specific rules for 352 of the 637 genes and constitutive expression for the remaining 285. Ninety-one unresolved TF signals were resolved through direct signal substitution, addition of new metabolite signals, or proxy replacement, producing a combined set of 1,643 Boolean rules. The rFBA procedure first solves a wild-type FBA to obtain flux signals, then evaluates all rules to determine gene ON/OFF states, propagated to reactions via GPR logic. Seven reactions required relaxation to preserve model viability. Across 46 aerobic carbon sources, rFBA identified 303 unique regulatory OFF reactions, and the constrained model achieves biomass of 0.6919 h ⁻ ¹ on glucose versus 0.6923 h ⁻ ¹ without regulatory constraints. The rFBA growth summary across all four baseline conditions and the 46 carbon-substrate conditions is reported in S1 Table, and the per-substrate list of reactions inactivated by regulatory rules and list of relaxed reactions is provided in S2 Table.

### 2.3. Single-gene knockout designs

The computational prediction of gene essentiality under various carbon substrate uptake relies on the calculation of maximum biomass formation in the presence of gene knockouts leading to the reaction eliminations implied by the Gene-Protein-Reaction (GPR) association rules. Their resolution relies on the solution of an optimization problem. Stating the formulation of the optimization problem requires the definition of the following sets:


$$\begin{array}{c} I=\left\{i|i=\text{1},\text{2},\ldots ,N\right\}\text{ set of metabolites} \end{array}$$

$$\begin{array}{c} J=\left\{j|j=\text{1},\;\text{2},\;\ldots ,M\right\}\text{ set of reactions} \end{array}$$

$$\begin{array}{c} G^{E.coli}=\left\{k\right|k=\text{1},\;\text{2},\;\ldots ,\;K\}\text{ set of genes in }E.\;coli \end{array}$$

$$\begin{array}{c} C=\left\{l|l=\text{1},\text{2},\;\ldots ,\;L\right\}\text{ set of substrates explored} \end{array}$$

where I and J are the set of metabolites and reactions in the metabolic network and $G^{E.coli}$ is the set of genes in *E. coli* metabolic network whose knockout effect would be explored. Furthermore, C represents the set of carbon substrates explored in this study. The complete list of these substrates is provided in Table 1. Metabolite steady state balance implies that,

**Table 1:** List of substrates analyzed in the study, selected from the Biolog database (http://www.biolog.com), all of which support *E. coli* growth in vivo.

Explored Substrates					
1	5-Keto-d-gluconic Acid	16	Glucose-6-Phosphate	31	Maltose
2	N-Acetyl-d-Glucosamine	17	d-Galactose	32	Maltotriose
3	N-Acetyl-Neuraminic Acid	18	Mucic Acid	33	d -Mannose
4	Adenosine	19	d -GalactonicAcid-γ-Lactone	34	d -Melibiose
5	α-Keto-GlutaricAcid	20	l -GalactonicAcid-γ-Lactone	35	d -Mannitol
6	d -Alanine	21	d -GalacturonicAcid	36	Pyruvic Acid
7	β- d -Allose	22	α- d -Glucose	37	d -Ribose
8	l -Arabinose	23	d -Gluconic Acid	38	l -Rhamnose
9	l -Aspartic Acid	24	d -Saccharic Acid	39	d -Sorbitol
10	2-DeoxyAdenosine	25	d -Glucuronic Acid	40	d -Serine
11	Fructose-6-Phosphate	26	Inosine	41	l -Serine
12	d -Fructose	27	l -Lactic Acid	42	Succinic Acid
13	l -Fucose	28	α- d -Lactose	43	Thymidine
14	Fumaric Acid	29	d -Malic Acid	44	d -Trehalose
15	Glucose-1-Phosphate	30	l -Malic Acid	45	Uridine
				46	d -Xylose


$$\begin{array}{c} \sum_{j\in J}s_{ij}v_{j}=\text{0},\;\forall i\in I \end{array}$$

where $s_{ij}$ is the stoichiometric coefficient of the metabolite i in reaction j and $v_{j}$ denotes the flux of the reaction j. For each gene k∈G, $D^{k}\subset J\;$is defined as the set of reactions that become inactive either because of the deletion of gene k as determined by the Gene-Protein-Reaction (GPR) rules, or through regulatory effects as determined by the evaluation of regulatory rules in the metabolic network. The set $D^{k}$ is specific to each gene deletion scenario and is updated accordingly when iterating through the set of genes G. To determine the maximum biomass formation under a specific substrate uptake condition l∈C, while accounting for the knockout of gene k set $U^{l}$ is defined as follows.

$U^{l}\subseteq J=\left\{j\in J\;\right|\;j\text{ is the uptake rxn for substrate}$ l∈C}

The following linear programming problem referred to as maxBiom, is formulated:

$\begin{array}{c} Maximize\;v_{biomass}\;[maxBiom] \end{array}$


$$\begin{array}{c} s.t \end{array}$$

$$\begin{array}{c} \sum_{j\in J}s_{ij}v_{j}=\text{0}\;\forall i\in I \end{array}$$

$$\begin{array}{c} LB_{j}\leq v_{j}\leq UB_{j}\;\forall j\in J \end{array}$$

$$\begin{array}{c} v_{j}=\text{0}\;j\in D^{k}\subset J \end{array}$$

$$\begin{array}{c} v_{j}=\text{1}\text{0}\;\forall j\in U^{l} \end{array}$$

$$\begin{array}{c} v_{o_{\text{2}}}\geq \text{2}\text{0} \end{array}$$

$$\begin{array}{c} v_{ATPM}=\overset{maint.}{\underset{ATPM}{v}} \end{array}$$

$$\begin{array}{c} v_{j}\in \;R\;\forall j\in J \end{array}$$

where, $v_{biomass}$ refers to biomass flux while $\overset{maint.}{\underset{ATPM}{v}}\;$ refers to growth associated maintenance ATP for maintenance. To ensure that all physiologically relevant metabolic flux values are captured, the upper and lower bounds, $UB_{j}$ and $LB_{j}$ were chosen accordingly. The value of upper bound for all reactions were set to 1000. The lower bound was set to zero for irreversible reactions and to -1000 for reversible reactions. To ensure metabolizing only one substrate at a time the uptake rate of all other substrate is set to zero and standardized uptake rate of 10 $mmol\;gDW^{-\text{1}}h^{-\text{1}}\;$was established for target carbon substrate, with the oxygen uptake rate set at 20 $mmol\;gDW^{-\text{1}}h^{-\text{1}}$ to reflect aerobic conditions. The non-growth associated ATP maintenance was also fixed at 8.39 $mmol\;gDW^{-\text{1}}h^{-\text{1}}$ [29].

By solving optimization problem (maxBiom) across different gene deletion and substrate uptake conditions, we can systematically assess gene essentiality. The results are organized into an Essentiality Score ($ES_{l,k}$) matrix, which is a binary matrix of dimension L×K, where L is total number of substrates exploring and K is total number of gene knockout. Each element $ES_{lk}$in the matrix is defined as:


$$\begin{array}{c} ES_{l,k}=\left\{ \begin{array}{c} @l\text{1}\text{ if gene k}\in \text{G is essential for growth on substrate l}\in \text{C }\\ \;\\ \text{0}\text{ otherwise} \end{array}\right. \end{array}$$

Each element in the ES matrix indicates whether a gene deletion mutant k can grow on substrate l ($i.e.,\;ES_{l,k}=\text{0}$) or not ($i.e.,\;ES_{l,k}=\text{1}$), thereby determining whether a gene is essential for growth under specific carbon substrate conditions. From this matrix, we can define a set of conditionally essential gene for each substrate $l,\;{CE}^{l}\;\subseteq G^{E.coli}$, which contains genes that are conditionally essential - that is, essential for growth on substrate l but not essential when growing on glucose:


$$\begin{array}{c} CE^{l}=\left\{k\in G\right|ES_{l,k}=\text{1}\;and\;{ES}_{glucose,\;k}=\text{0}\} \end{array}$$

These conditionally essential gene sets enable systematic identification of potential auxotrophic strains for each substrate. Genes in $CE^{l}$ set are non-essential for growth on glucose but become essential when cells are switched to substrate l, making them ideal candidates for designing substrate-specific auxotrophs. For each identified conditionally essential gene k in $CE^{l}$, potential rescue mechanisms can be determined by solving an optimization problem that restores growth through the addition of candidate functions. Rescue either re-enables flux through a knocked-out *i*ML1515 reaction or introduces a reaction from the digital reaction library, depending on whether the required function is already present in the model. In both cases the rescued reaction receives an upper flux bound of 1,000 mmol gDW ⁻ ¹ h ⁻ ¹, with all other substrate, regulatory, and knockout constraints unchanged. GPR associations are not modified, as the objective is to determine which metabolic function restores growth rather than which gene performs it; gene-function assignment is reserved for the experimental complementation step.

By defining following set:

$U^{ext}=\left\{j^{\prime }\right|j^{\prime }=\text{1},\;\text{2},\;\ldots ,\;J^{\prime }\}\text{ set of non-E. coli reactions from external database}$

where $U^{ext}$ is the set of reactions that are candidates for restoring growth drawn from a digital reaction library comprising external reactions from the BiGG universal [41]  and MetaNetX universal models [42]. For each gene ${k^{\prime }\in G}^{Database}$, $D^{k^{\prime }}\;$can be defined as a set of reactions that become active because of GPR rules associated with adding gene $k^{\prime }$ to the mutant. For each gene deletion mutant (k), where $D^{k}$ represents its set of inactivated reactions, the ability of a rescue gene ($k^{\prime }$) to restore growth on a specific substrate (l∈C) is determined through the following optimization problem:


$$\begin{array}{c} Maximize\;\overset{mutant}{\underset{biomass}{v}}\;[ResMut] \end{array}$$

$$\begin{array}{c} s.t \end{array}$$

$$\begin{array}{c} \sum_{j\in J}s_{ij}v_{j}=\text{0}\;\forall i\in I \end{array}$$

$$\begin{array}{c} LB_{j}\leq v_{j}\leq UB_{j}\;\forall j\in J \end{array}$$

$$\begin{array}{c} v_{j}=\text{0}\;j\in D^{k}\;\subset J \end{array}$$

$$\begin{array}{c} v_{j}\geq \;\text{1}\text{0}\text{0}\text{0}\;j\in D^{k^{\prime }} \end{array}$$

$$\begin{array}{c} v_{j}=\text{1}\text{0}\;\forall j\in U^{l} \end{array}$$

$$\begin{array}{c} v_{o_{\text{2}}}\geq \text{2}\text{0} \end{array}$$

$$\begin{array}{c} v_{ATPM}=\overset{maint.}{\underset{ATPM}{v}} \end{array}$$

$$\begin{array}{c} v_{j}\in \;R\;\forall j\in J \end{array}$$

The solution to ResMut defines growth restoration as successful when the optimal biomass formation is more than 10% of the wild-type growth. For each $k\in CE^{l}\subseteq G$, the solution in ResMut identifies candidate genes ${k^{\prime }\in G}^{Database}$, that restore growth when added to the mutant strain. This systematic evaluation yields a collection of auxotrophic designs characterized by a single gene deletion k from $CE^{l}$ causing substrate-specific growth defects and identified rescue genes $k^{\prime }$ from $G^{Database}$, that restore growth functionality. These substrate-specific auxotrophic strains serve as tools for functional annotation of uncharacterized ORFs, enabling the identification of both transport and enzymatic functions based on their growth rescue patterns. All single-gene KO designs and their rescue functions are listed in S3 Table.

### 2.4. Double gene knockout designs

The gene deletion analysis can be extended to double gene deletions by modifying the original formulation. For each substrate l, a set of viable double gene deletions ${G\text{2}}^{l}$is defined as:


$$\begin{array}{c} {G\text{2}}^{l}=\left\{\left(k_{\text{1}},\;k_{\text{2}}\right)\right|\;k_{\text{1}},\;k_{\text{2}}\in G^{E.coli}\backslash CE^{l},\;k_{\text{1}}\text{≠}k_{\text{2}}\} \end{array}$$

where $G^{E.coli}\backslash CE^{l}$ denotes the set of all genes in the model excluding those in single essential genes for each substrate ($CE^{l})$. The maxBiom problem can then be solved for each gene pair in ${G\text{2}}^{l}$, leading to a new essentiality matrix ES2. Similar to the single gene deletion matrix ES, ES2 is a binary matrix where rows represent substrates l ∈ C, but columns now represent gene pairs from ${G\text{2}}^{l}$. Each element $ES{\text{2}}_{l,(k\text{1},k\text{2})}$ indicates whether the double deletion mutant can grow (0) or not (1) on substrate l. For each identified non-growing double deletion mutant, potential rescue mechanisms are determined using the same optimization framework applied to single-gene deletions. The rescue problem (ResMut) can be solved for each candidate rescue gene ${k^{\prime }\in G}^{Database}$, where successful growth restoration is defined as achieving biomass production exceeding 10% of wild-type growth. This expanded analysis identifies more complex metabolic dependencies and gene function interactions that may not be evident from single gene deletion studies, while also revealing potential rescue mechanisms that can restore growth in these more complex genetic backgrounds. The full set of conditionally essential gene pairs is provided in S4 Table, and the corresponding rescue functions in S5 Table.

### 2.5. Triple gene knockout designs

The gene deletion analysis was further extended to triple-gene knockouts to discover pathways buffered by three or more isozymes or paralogous functions. Because the combinatorial space of all possible gene triples is prohibitively large for a 1,516-gene model, screening was restricted to genes not essential on glucose, with each candidate triple further evaluated against three filters: (i) no individual gene *k*ᵢ is conditionally essential alone on substrate *l*; (ii) no gene pair within the triple constitutes a double synthetic lethal on substrate *l*; and (iii) the triple knockout biomass falls below 10% of wild-type growth. This ensures that identified triples are irreducible three-way synthetic-lethal combinations with no lower-order sub-component. Rescue analysis was performed identically to single- and double-gene designs, querying the digital reaction library for functions that restore growth when all three knocked-out reaction sets are held at zero flux. All conditionally essential gene triples and their rescue functions are reported in S6 and S7 Tables, respectively.

All of the analysis described in this study was coded using the Constraint-Based Reconstruction and Analysis toolbox for Python (COBRApy version 0.13.3) [38] and IBM ILOG CPLEX solver [40] for all linear programming LP problems. All scripts and functions were run in the Python 3 environment (version 3.10.13) [43].

### 2.6. Experimental setup

To verify the computational predictions and assess potential bypass pathways compensating for deleted genes, growth phenotypes were tested under defined conditions. Overnight cultures of knockout strains from the Keio collection were prepared, and 5 μL of each culture was inoculated into 500 μL of M9 minimal media supplemented with 4 g/L of the predicted non-utilizable carbon source in a 24-well plate. Cultures were incubated in a Spark multimode microplate reader at 37 °C for 24 hours, and OD₆₀₀ was measured at regular intervals to assess growth. Glucose (4 g/L) was used as a positive control, while M9 media without a carbon source served as the negative control. These tests were designed to assess the accuracy of model predictions and reveal potential metabolic bypass mechanisms in the knockout strains.

Growth analysis was performed using water corrected metrics to account for background optical density changes. For each strain and substrate combination, growth was calculated as


$$\begin{array}{c} \Delta ln(OD_{\text{6}\text{0}\text{0}})\;=\;ln(O\overset{final}{\underset{\text{6}\text{0}\text{0}}{D}})\;-\;ln(O\overset{initial}{\underset{\text{6}\text{0}\text{0}}{D}}) \end{array}$$

where $O\overset{final}{\underset{\text{6}\text{0}\text{0}}{D}}\;$and $O\overset{initial}{\underset{\text{6}\text{0}\text{0}}{D}}$ represent the last non-null and first non-null $OD_{\text{6}\text{0}\text{0}}$ measurements during exponential phase, respectively. Net growth on substrate was calculated as


$$\begin{array}{c} \Delta {ln}_{sub}=\;\Delta ln{\left(OD_{\text{6}\text{0}\text{0}}\right)}_{substrate}-\;\Delta ln{\left(OD_{\text{6}\text{0}\text{0}}\right)}_{water} \end{array}$$

and net growth on glucose was calculated as


$$\begin{array}{c} \Delta {ln}_{glu}=\;\Delta ln{\left(OD_{\text{6}\text{0}\text{0}}\right)}_{glucose}\;-\;\Delta ln{\left(OD_{\text{6}\text{0}\text{0}}\right)}_{water} \end{array}$$

Fractional growth was defined as the following ratio


$$\begin{array}{c} \phi =\frac{{\Delta ln}_{sub}}{\Delta {ln}_{glu}} \end{array}$$

representing the proportion of glucose-level growth achieved on the test substrate.

Phenotype classification was performed using a two-parameter criterion system. Strains were classified as exhibiting growth (false positive prediction) if


$$\begin{array}{c} {\Delta ln}_{sub}\geq \;\text{0}.\text{6}\text{0}\;AND\;\phi \;\geq \;\text{0}.\text{5}\text{5} \end{array}$$

indicating robust growth comparable to glucose controls. Strains were classified as exhibiting slight growth (partial auxotrophy) if


$$\begin{array}{c} {\Delta ln}_{sub}\geq \;\text{0}.\text{1}\text{0}\;AND\;\phi \;\geq \;\text{0}.\text{2}\text{0} \end{array}$$

indicating intermediate growth between auxotrophy and wild type. Strains failing to meet these thresholds were classified as exhibiting no growth (validated auxotrophy). This dual parameter approach accounts for both absolute growth magnitude and relative performance compared to glucose, providing more robust phenotype assignment than single-threshold methods. Sensitivity analysis was performed by varying $\phi_{low}$(0.15, 0.20, 0.25), $\phi_{high}$ (0.50, 0.55, 0.60), and $\Delta ln_{sub}$ (0.05, 0.10, 0.15). Across all threshold combinations tested, approximately 10 of 61 strains (16%) changed classification, confirming that the reported auxotrophy rates are robust to reasonable variation in these cutoffs. Phenotype classifications, the threshold sensitivity grid, and the borderline strains are reported in S8 Table; the time-resolved OD_600_ traces underlying these values are provided in S9 Table and visualized as growth-curve panels in S1 Fig. Analysis scripts are archived in the project repository.

## 3. Results

In this study, a systematic computational approach was developed to design conditional auxotrophic strains through strategic single, double, and triple gene deletions across diverse carbon substrate conditions. Comprehensive gene essentiality simulations, encompassing both single and double gene deletions, were performed to identify conditionally essential genes and their potential rescue mechanisms. This systematic analysis across 46 different carbon substrates led to the computational design of 258 unique (97 single genes, 142 gene pairs, and 19 gene triples) auxotrophic strains in *E. coli*. These engineered strains exhibit normal growth on glucose but require specific genetic and metabolic complementation when utilizing alternative carbon substrates.

**Fig 3:**
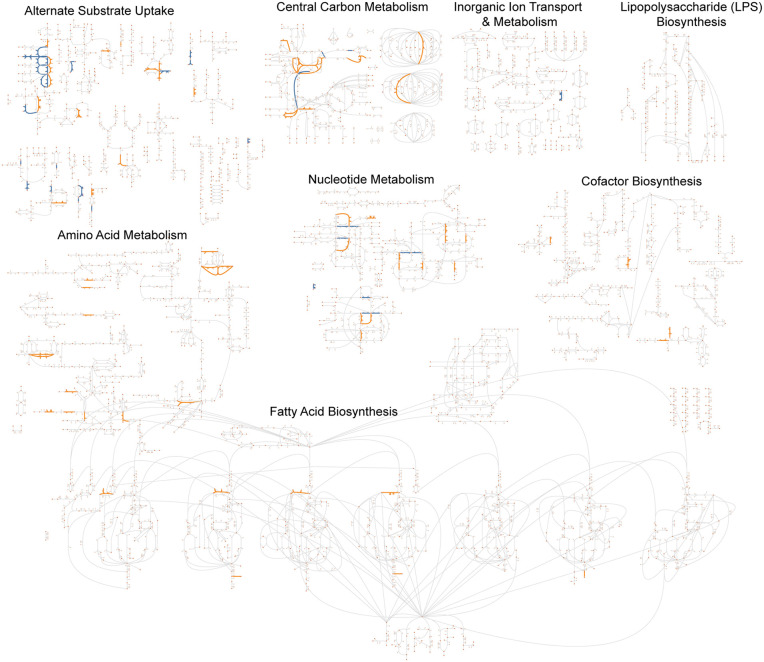
Bird's-eye view map, Escher network map of *E. coli* showing in blue all transport function and orange all enzymatic functions identified by this analysis to demonstrate the comprehensive scope and coverage of metabolic functions explored in this study. The majority of the designs are strategically situated within the central carbon metabolism and other critical carbon metabolic pathways, underscoring the focal areas of our genetic manipulations. This map illustrates how various segments of the metabolic network are covered in terms of functional outcomes resulting from our designs. Reactions (lines) connect metabolites (circles).

The designed strains provide a platform for functional genomics, particularly for elucidating the roles of uncharacterized open reading frames (ORFs). Through systematic analysis, a total of 258 designs is supported by 220 cumulative unique rescue functions across all three knockout tiers, of which 56 correspond to transport functions and 164 to enzymatic functions. Of the single-gene designs, 70 are maximally specific, requiring exactly one rescue function and thus providing unambiguous phenotype-to-function mapping. The double-gene designs expand the functional scope with 120 rescue functions exclusively accessible via synthetic lethal combinations, covering biosynthetic and cofactor pathways unreachable by single-gene knockouts. A further 11 rescue functions are exclusively accessible via triple-gene designs, representing pathways buffered by three independent isozymes or transporter paralogs including DAHP synthase, ferrochelatase, and inorganic pyrophosphatase. Fig 3 provides a bird’s-eye view of all functions that can be resolved using the designed auxotrophic strains, visualized on the *E. coli* metabolic network. The functional distribution spans critical areas including transport systems, central carbon metabolism, alternative substrate utilization pathways and nucleotide metabolism, demonstrating the comprehensive coverage achieved by this approach.

The auxotrophic designs developed in this study can be strategically applied for genotype-phenotype mapping through two distinct approaches. The first category enables direct one-to-one mapping, where growth restoration upon complementation immediately reveals the function of an uncharacterized ORF. The second category comprises more complex cases where growth can be restored through multiple functional complementation, requiring sequential testing to precisely determine the underlying gene function. This dual approach provides a comprehensive framework for systematic functional genomics studies. All single-, double-, and triple-gene knockout designs and their rescue functions are reported in S3, S5 and S7 Tables, respectively. A complete index of supporting information files is provided at the end of the manuscript. Intermediate pipeline outputs (gene ON/OFF activity matrix, baseline-condition OFF reaction counts, the digital reaction library, and pre-deduplication rescue variants) are archived in the project repository at https://github.com/maranasgroup/auxotroph-design.

### 3.1. Gene essentiality analysis

A comprehensive gene essentiality analysis was performed across 46 different substrates in *E. coli*, encompassing both single and double gene deletions. The workflow, detailed in the Methods section, evaluated the essentiality of 1,516 genes in *i*ML1515 genes to identify candidates for auxotrophic strain design. The analysis revealed distinct essentiality patterns across different carbon sources. Table 2 demonstrates the number of conditionally essential genes for each substrate, genes that become essential when growing on substrates other than glucose. For instance, mutant ∆*srl* can grow on glucose but becomes essential when d-sorbitol is the sole carbon source, as *SrlA* is involved in sorbitol transport but does not participate in the glucose utilization pathway.

**Table 2:** Carbon substrates grouped by number of conditionally essential genes.

No. of Genes	Carbon Substrates
16	Succinic Acid
14	d -Malic Acid
13	d -Alanine, d-Serine
12	l -Aspartic Acid, l -Lactic Acid, l-Serine, Pyruvic Acid
11	Fumaric Acid, l -Malic Acid
8	l -Rhamnose, Maltotriose
7	d -Mannitol, l -Fucose, Maltose
6	d -Fructose, d -Mannose, d -Sorbitol, d -Trehalose, l-Galactonic Acid, Mucic Acid, N-Acetyl-Neuraminic Acid, β-d -Allose
5	d -Glucuronic Acid, d -Melibiose, Glycerol, α-Keto-Glutaric Acid
4	2-DeoxyAdenosine, d -Galactonic Acid, d -Saccharic Acid, N-Acetyl-d-Glucosamine
3	d -Galactose, Thymidine
2	5-Keto- d -Gluconate, l -Arabinose
1	Adenosine, d -Ribose, d -Xylose, Fructose-6-Phosphate, Glucose-6-Phosphate, Inosine, Lactose, Uridine

The computational predictions for conditional essentiality were validated against experimental results from literature [2], demonstrating 93% accuracy in predicting gene essentiality under different substrate uptakes.

A comprehensive double gene deletion analysis was also performed across all carbon substrates to identify additional functionalities beyond those revealed by single gene knockouts. S4 Table lists all gene pairs that become essential when growing on different substrates. The analysis identified 3,823 conditionally essential gene pairs across 46 substrates, involving 201 unique genes in 288 unique gene pair combinations. Of these pairs, 2,333 (*i.e.*, 61%) yielded at least one rescue function, with 142 unique rescue functions identified in total. To quantify the added value of double-gene designs, the rescue functions identified by double-gene knockouts were compared to those accessible by single-gene knockouts. Of the 142 DKO rescue functions, 120 (*i.e.*, 85%) are exclusively accessible via double-gene knockouts and cannot be detected by any single-gene design. These DKO-exclusive functions systematically cover biosynthetic and cofactor pathways where single-gene knockouts are lethal under glucose conditions: pyrimidine biosynthesis (carbamoyl phosphate synthetase, CTP synthetase), one-carbon and serine metabolism (glycine hydroxymethyltransferase, phosphoserine aminotransferase), aromatic amino acid biosynthesis (shikimate kinase), arginine biosynthesis (ornithine carbamoyltransferase), heme biosynthesis (coproporphyrinogen oxidase), and sulfur amino acid biosynthesis (cysteine synthase). For example, while Δ(araB, xylB) double mutant grows normally on glucose, it becomes non-viable when xylose is the sole carbon source, as both genes must be deleted to eliminate xylokinase activity. Notably, the molybdate (*modA*, *modB*, *modC*) and sulfate (*cysA*, *cysW*, *cysU*) transporter subunits are exclusively synthetic lethal with each other: every pairwise combination of one gene from each family eliminates growth across all 46 tested non-glucose substrates, reflecting a universal metabolic dependency on simultaneous molybdenum cofactor and sulfur supply.

Triple-gene knockout analysis identified 375 conditionally essential combinations across 45 substrates, involving 66 unique gene triples and 49 genes. Seven triples are conditionally essential across all 45 substrates, revealing universal metabolic dependencies. The *aroG* + *aroH* + *aroF* triple eliminates all three feedback-regulated DAHP synthase isozymes, blocking the shikimate pathway under any growth condition. The *hemH* + *efeB* + *yfeX* triple probes ferrochelatase redundancy, creating a selective condition to resolve the debated in vivo contribution of the partially characterized *efeB* and *yfeX* to heme biosynthesis. Three further triples target pyrophosphate clearance (*ppx* + *umpG* + *ppa*), undecaprenyl-PP phosphatase (*ybjG* + *pgpB* + *bacA*), and molybdate/sulfate transport cross-reactivity (*modABC* + *cysP* + *sbp*). The remaining 59 substrate-specific triples include all three fumarase isozymes (*fumA* + *fumB* + *fumC*) on fumaric and succinic acids, all three C4-dicarboxylate transporters (*dcuA* + *dcuB* + *dcuC*) on l-malic acid, and 48 triples on d-ribose arising from combinations of the characterized ribose (*rbsABCD*) and allose (*alsABC*) ABC transporters with four uncharacterized subunit genes (*ytfQ*, *ytfR*, *ytfT*, *yjfF*), predicting alternative ribose transport functions for these unannotated ORFs. Rescue analysis identified 11 novel rescue functions exclusive to this tier, with 373 of 374 records achieving 100% wild-type biomass restoration (S6 Table).

The identified conditionally essential genes, from both single and double deletion analyses, were systematically evaluated for growth rescue through genetic complementation as described in the Methods section. This complementation analysis revealed specific functions capable of restoring growth under restrictive conditions. For single gene deletions, the rescue of growth through complementation directly indicated the function of the deleted gene. For double gene deletions, the restoration of growth often revealed more complex functional relationships, including cases of redundancy and parallel pathways. The comprehensive mapping of these growth rescue patterns provided a framework for systematic function discovery. In the following sections, we detail how this complementation patterns can be strategically applied to elucidate the function of uncharacterized open reading frames (ORFs), presenting both straightforward cases where single complementation directly reveals function and more complex scenarios requiring multiple complementation approaches.

### 3.2. One-to-one auxotrophic designs

A subset of the designed auxotrophic strains enables direct one-to-one mapping between strain growth and specific rescuing functions. Among the 258 total auxotrophic designs, 128 strains exhibit this one-to-one mapping characteristic, with 39 strains corresponding to transport functions and 89 to enzymatic functions. These designs comprise 70 single gene knockouts and 41 double gene knockouts and 17 triple gene knockouts, each providing unambiguous functional assignments. S3 Table presents representative examples of these designs, including the knocked-out genes, the corresponding auxotrophic substrates, and the specific functions required for growth restoration. Equivalent designs for double- and triple-gene knockouts are reported in S5 and S7 Tables respectively.

One-to-one mapping results provide a direct connection between growth rescue and gene function. For transport functions, the rescue patterns strongly suggest expanded substrate specificity, while for enzymatic functions, they point at secondary enzymatic functions. For example, model prediction suggests that when gene *araA* (b0062) is deleted, the strain cannot grow on l-arabinose without complementation the activity but grows on glucose.

**Fig 4:**
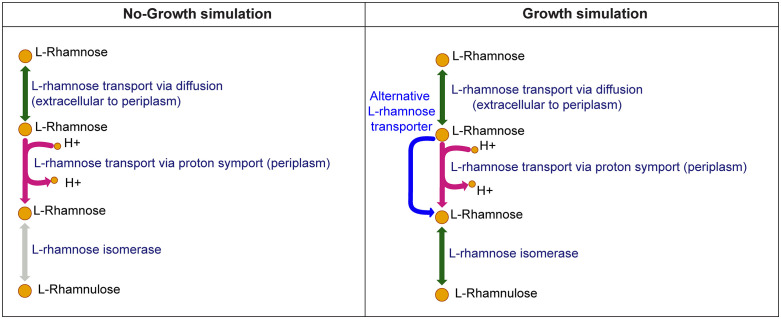
Illustration of an auxotrophic strain with a self-replacement transporter functionality, where the deleted function is complemented by a unique function (i.e., l-rhamnose transporter) to restore growth. The strain can grow on glucose but becomes auxotrophic when the conditionally essential gene (rhaT) is deleted, affecting its ability to grow on l-rhamnose. Green arrows represent reactions carrying flux, magenta arrows indicate reactions impacted by the gene knockout, and blue arrows denote the added functionality required to restore growth.

Also, Fig 4 shows a targeted one-to-one complementation strategy designed to investigate l-rhamnose transport by comparing growth in a single-gene deletion mutant (∆*rhaT*) of *E. coli* under two scenarios: the “no-growth simulation” (left) and the “growth simulation” (right). In the no-growth simulation, disabling gene *rhaT* prevents efficient proton symport of l-rhamnose from the periplasm into the cytoplasm, halting rhamnose metabolism at the isomerization step and prohibiting cell growth when rhamnose is the sole carbon source. In contrast, the growth simulation panel demonstrates that reintroducing or substituting the transporter restores l-rhamnose uptake, allows for normal progression through the rhamnose metabolic pathway and enables robust cell growth. This targeted deletion–complementation approach underscores how *RhaT* directly governs rhamnose utilization, since no alternative pathways compensate for the transporter’s function in the knockout strain. By examining growth outcomes only when RhaT (or an alternative transporter) is reintroduced, researchers can isolate the precise contribution of rhamnose transport to overall metabolism and further explore auxiliary factors such as regulation, transporter specificity, and evolutionary adaptations in l-rhamnose utilization.

**Fig 5:**
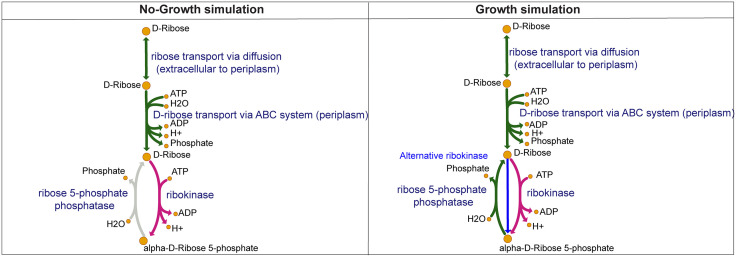
Illustration of an auxotrophic strain with a self-replacement enzymatic functionality, where the deleted function is complemented by a unique function (i.e., ribokinase) to restore growth. The strain can grow on glucose but becomes auxotrophic when the conditionally essential gene (rbsK) is deleted, affecting its ability to grow on d-ribose. Green arrows represent reactions carrying flux, magenta arrows indicate reactions impacted by the gene knockout, and blue arrows denote the added functionality required to restore growth.

Moreover, Fig 5 applies a similar deletion–complementation approach for ribokinase. Here, knocking out the ribokinase gene hinders the phosphorylation of d-ribose to α- d-ribose 5-phosphate, leading to a no-growth scenario on ribose as the sole carbon source (left panel). Restoring ribokinase (or providing an alternative) reinstates this critical phosphorylation step (right panel), thereby allowing downstream metabolism to proceed and supporting cell proliferation. By pinpointing ribokinase’s essential contribution, this design opens avenues for examining regulatory interactions, engineering novel kinases, and optimizing sugar utilization in biotechnological applications.

### 3.3. More complex designs

Some auxotrophic designs can be complemented by more than one function, making the identification of the function of the added ORF more complex compared to straightforward self-replacement designs. In these cases, multiple functions may restore growth in the strain, meaning that the mapping between the added ORF and the missing function is not unique. As a result, these designs require additional steps, such as performing more than one growth/no-growth test, to accurately determine the unknown function associated with the ORF.

These auxotrophic designs can be useful for studying pathways or genes with overlapping or redundant roles in metabolic networks. For example, in cases where multiple enzymes or transporters contribute to the same pathway, the deletion of a single gene may not completely disrupt flux until additional constraints are imposed. Similarly, regulatory genes or structural components may have indirect roles, leading to more than one possible rescue mechanism. This highlights the importance of systematically testing multiple experimental conditions to resolve ambiguities in function assignment.

**Fig 6:**
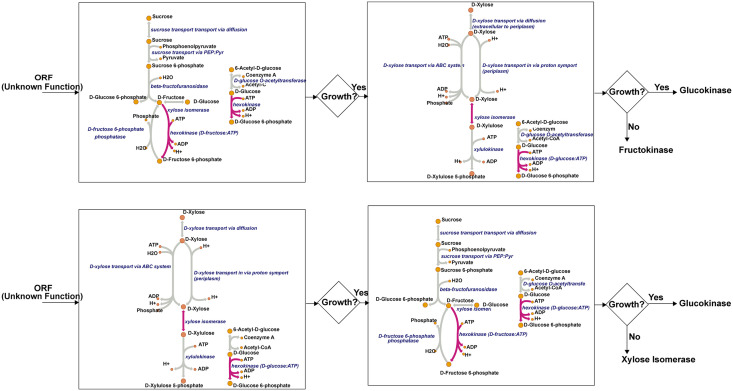
Systematic functional characterization of an unknown ORF using dual synthetic lethal designs. The figure illustrates two parallel experimental approaches (top and bottom panels) to determine the function of an uncharacterized ORF. Each design tests the ORF against different enzyme pairs: Design 1 (top) examines glucokinase/fructokinase, while Design 2 (bottom) tests glucokinase/xylose isomerase combinations. In both designs, growth (“Yes”) was observed only in strains requiring glucokinase activity, while no complementation (“No”) occurred for fructokinase or xylose isomerase functions. The consistency across both designs provides strong evidence that the unknown ORF encodes a protein with glucokinase activity. Red markings in the metabolic maps highlight the relevant pathway sections under investigation.

As an example, Fig 6 demonstrates a complex functional mapping approach using synthetic lethal designs to characterize an unknown ORF’s function. Two independent experimental designs were employed, each testing the ORF against different enzyme combinations: glucokinase with fructokinase (Design 1) and glucokinase with xylose isomerase (Design 2). In both designs, when the unknown ORF was present, cell growth was observed (“Yes” outcomes), specifically in strains requiring glucokinase activity. Conversely, the ORF failed to complement strains requiring either fructokinase or xylose isomerase function (“No” outcomes). This consistent pattern across multiple synthetic lethal combinations provides strong evidence that the uncharacterized ORF encodes a protein with glucokinase activity. The power of this approach lies in its use of multiple, independent synthetic lethal combinations to triangulate the specific function of the unknown gene. By testing against different enzyme pairs and observing which functions can and cannot be complemented, researchers can make robust conclusions about the ORF’s functional role. This method can in principle uncover functional information more reliably through multiple independent lines of genetic evidence.

Furthermore, these multi-functional designs emphasize the flexibility and robustness of the computational approach, as they capture complex dependencies that would otherwise go unnoticed in simpler models. They could serve as a foundation for studying gene interactions and exploring alternative metabolic routes, providing insights into how *E. coli* and other organisms adapt to environmental challenges through pathway redundancy or compensatory mechanisms. These designs, while less direct, may play an important role in refining functional annotations and uncovering deeper layers of metabolic complexity.

### 3.4. Characterization of predicted rescue functions

The 220 cumulative unique rescue functions span two categories, 56 transport (25%) and 164 enzymatic (75%) reactions. At the SKO tier, 89 unique rescue functions are identified; 70 of the 97 SKO strains (*i.e.*, 72%) are maximally specific. The DKO tier, which adds 120 exclusive rescue functions that cannot be achieved through a single knockout, demonstrates that combinatorial knockouts systematically uncover functional space that single-deletion screening cannot access. The TKO tier adds 11 further exclusive functions, for 19 TKO total. Rescue potential is high, with 98.7% of SKO rescue events and 373 of 374 TKO rescue records achieving 100% wild-type biomass recovery.

### 3.5. Experimental validation of computational predictions

**Fig 7:**
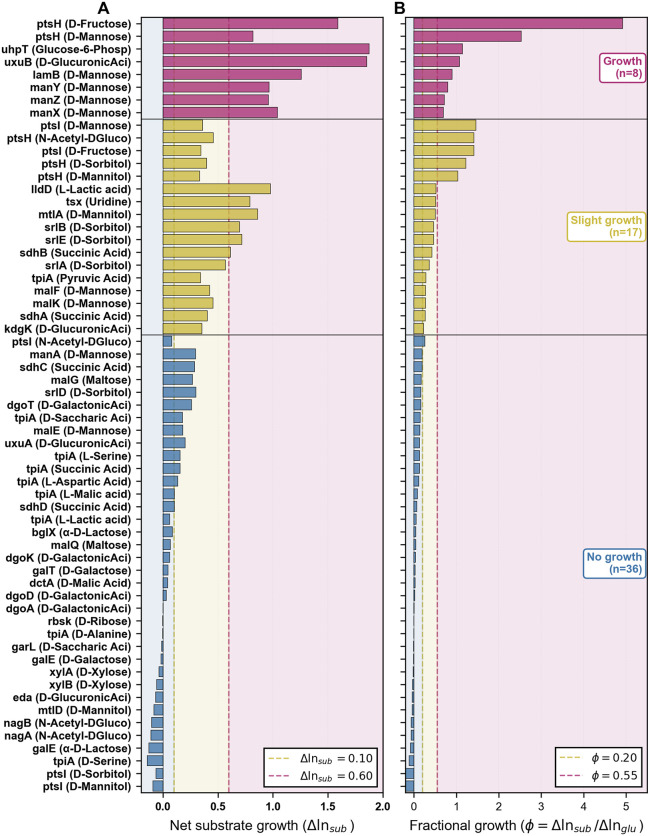
Horizontal bar charts displaying net substrate growth ($\Delta ln_{sub}$, Panel A) and fractional growth (ϕ, Panel B) for all 61 tested strain-substrate combinations. Strains are sorted by classification category and fractional growth value within each category. Bars are color-coded by classification: validated auxotrophs (blue, $\phi \;<\;\text{0}.\text{2}\text{0}\;or\;\Delta ln_{sub}<\;\text{0}.\text{1}\text{0},\;n=\text{3}\text{6}$), partial auxotrophs (yellow, meeting minimum thresholds but not growth criteria, n=17), and false positives (purple, $\phi \;\geq \;\text{0}.\text{5}\text{5}\;and\;\Delta ln_{sub}\geq \;\text{0}.\text{6}\text{0},\;n=\text{8}$). Vertical dashed lines indicate classification thresholds: $\Delta ln_{sub}\;=\;\text{0}.\text{1}\text{0}$ (yellow) and 0.60 (purple) in Panel A; ϕ = 0.20 (yellow) and 0.55 (purple) in Panel **B.** Background shading indicates classification zones. Each bar represents the water-corrected growth measurement over 24 hours. Both metrics are dimensionless. The dual-panel layout emphasizes the two-parameter nature of the classification system, where both criteria must be satisfied for growth or slight growth designation.

To assess the veracity of the computational framework, the predicted auxotrophic phenotypes for a subset of designed strains were experimentally tested. This entailed confirming that computationally designed gene deletions indeed create substrate-specific growth defects as predicted and their growth profile. Sixty-one computationally designed single-gene auxotrophic strains were tested by measuring growth through optical density measurements over 24 hours. Growth analysis was performed using water-corrected metrics as described in Methods, quantifying net substrate growth ($\Delta ln_{sub}$), net glucose growth ($\Delta ln_{glu}$), and fractional growth ($\phi \;=\;\frac{\Delta ln_{sub}}{\Delta ln_{glu}}$). This revealed three distinct phenotype categories based on the two-parameter classification system(Fig 7, Table 3). Of the 61 strains tested, 36 (59.0%) exhibited confirmed auxotrophy with negligible growth on their predicted auxotrophic substrates ($\phi \;<\;\text{0}.\text{2}\text{0}\;or\;\Delta ln_{sub}<\;\text{0}.\text{1}\text{0}$), 17 (*i.e.*, 27.9%) showed partial auxotrophy with intermediate growth ($\phi \;\geq \text{0}.\text{2}\text{0}\;and\;\Delta ln_{sub}\geq \text{0}.\text{1}\text{0}$), and 8 (*i.e.*, 13.1%) displayed robust growth indicating false auxotrophy model predictions ($\phi \;\geq \;\text{0}.\text{5}\text{5}\;and\;\Delta ln_{sub}\geq \;\text{0}.\text{6}\text{0}$) (S8 & S9 Tables, S1 Fig). The experimental validation reported here focused on single-gene knockout strains using the Keio collection, which provided immediate access to 61 pre-constructed deletion mutants spanning a broad range of substrates and metabolic functions. Validation of double and triple knockout designs, which require combinatorial strain construction, falls beyond the scope of the current study. Similarly, because the rescue complementation step was not experimentally performed, the 61 validated designs confirm auxotrophic phenotype but do not yet test the rescue predictions; the relationship between rescue function specificity and phenotype confirmation therefore awaits dedicated complementation experiments.

**Table 3:** Example of tested auxotrophic designs and their growth metrics. Net glucose growth ($\Delta ln_{glu}$) and net substrate growth ($\Delta ln_{sub}$) represent water-corrected ($ln_{OD_{600}}$) changes over 24 hours. Fractional growth (ϕ) indicates substrate growth as a proportion of glucose growth^1^.

Mutant	Alternative Substrate	$\Delta {ln}_{glu}\;$	${\Delta ln}_{sub}$	ϕ	Classification
Δ*galT*	d-Galactose	1.697	0.045	0.027	No growth
Δ*nagB*	N-Acetyl- d-Glucosamine	1.583	-0.105	-0.067	No growth
Δ*bglX*	α-d-Lactose	1.832	0.084	0.046	No growth
Δ*rbsK*	d-Ribose	1.773	-0.003	-0.002	No growth
Δ*srlD*	d-Sorbitol	1.802	0.298	0.165	No growth
Δ*dctA*	d-Malic Acid	1.731	0.042	0.024	No growth
Δ*sdhC*	Succinic Acid	1.466	0.286	0.195	No growth
Δ*sdhD*	Succinic Acid	1.451	0.103	0.071	No growth
Δ*dgoA*	d-Galactonic Acid-γ-Lactone	1.660	0.001	0.001	No growth
Δ*xylB*	d-Xylose	1.809	-0.059	-0.032	No growth
Δ*manA*	d-Mannose	1.492	0.296	0.198	No growth
Δ*srlB*	d-Sorbitol	1.501	0.697	0.464	Slight growth
Δ*sdhB*	Succinic Acid	1.437	0.613	0.427	Slight growth
Δ*mtlA*	d-Mannitol	1.711	0.861	0.503	Slight growth
Δ*lldD*	l-Lactic Acid	1.889	0.979	0.518	Slight growth
Δ*ptsH*	d -Fructose	0.323	1.592	4.924	Growth
Δ*uxuB*	d-Glucuronic Acid	1.730	1.854	1.071	Growth
Δ*manX*	d-Mannose	1.500	1.042	0.695	Growth

^1^Strains classified as “No growth” ($\phi \;<\;\text{0}.\text{2}\text{0}\;or\;\Delta ln_{sub}\;<\;\text{0}.\text{1}\text{0}$) are validated auxotrophs; “Slight growth” $\phi \;\geq \text{0}.\text{2}\text{0}\;and\;\Delta ln_{sub}\geq \text{0}.\text{1}\text{0}$) indicates partial auxotrophy; “Growth” ($\phi \;\geq \;\text{0}.\text{5}\text{5}\;and\;\Delta ln_{sub}\;\geq \;\text{0}.\text{6}\text{0}$) indicates false positive predictions.

Validated auxotrophs demonstrated severe growth defects with fractional growth values near zero, confirming that the deleted genes are indeed essential for growth on their respective substrates. Notable examples included *ΔgalT* on d-galactose (ϕ = 0.027), *ΔnagB* on N-acetyl-d-glucosamine (ϕ = −0.067), *ΔrbsK* on d-ribose (ϕ= −0.002), and *ΔbglX* on α-d-lactose (ϕ = 0.046). Complete agreement was observed for d-galactonic acid-γ-lactone utilization, with all four gene deletions (*ΔdgoA,* Δ*dgoD,* Δ*dgoK,* Δ*dgoT*) resulting in fractional growth values below 0.2. Similarly, xylose utilization genes (*ΔxylA*, *ΔxylB*) and key succinate dehydrogenase subunits (ΔsdhC, ΔsdhD) produced validated auxotrophic phenotypes.

Partial auxotrophs occupied an intermediate phenotype space, meeting minimum growth thresholds but showing substantial metabolic impairment compared to wild-type growth on glucose. Representative examples included *ΔsrlB* on d-Sorbitol ($\Delta ln_{sub_{net}}\;=\;\text{0}.\text{6}\text{9}\text{7},\;\phi \;=\;\text{0}.\text{4}\text{6}\text{4}$), *ΔsdhB* on succinate ($\Delta l{n_{sub}}_{net}\;=\;\text{0}.\text{6}\text{1}\text{3},\;\phi \;=\;\text{0}.\text{4}\text{2}\text{7}$), and *ΔmtlA* on d -mannitol ($\Delta l{n_{sub}}_{net}\;=\;\text{0}.\text{8}\text{6}\text{1},\;\phi \;=\;\text{0}.\text{5}\text{0}\text{3}$). Multiple *ΔtpiA* variants tested on organic acids displayed partial auxotrophy, with fractional growth values ranging from 0.084 to 0.280, suggesting that triose-phosphate isomerase deletion imposes metabolic constraints but permits low-level growth through alternative routes. Partial auxotrophy reflects low-level activity of alternative routes not fully captured in the model, representing refinement model targets.

False positive predictions include PTS component deletions with Δ*ptsH* and Δ*ptsI* maintaining substantial growth across multiple substrates (ϕ values of 1.41− 4.92), alluding to either extensive cross functionality among PTS components or uncharacterized alternative transport systems. The maltose/mannose ABC transporter components (Δ*lamB*, Δ*manX*, Δ*manY*, Δ*manZ*) achieved ϕ values of 0.69−0.90 on d-mannose, suggesting broader substrate specificity than currently present in the metabolic model. Additionally, deletion mutant Δ*uxuB* grown on d-glucuronic acid (ϕ = 1.071) and Δ*uhpT* on glucose-6-phosphate (ϕ = 1.145) displayed unexpected metabolic flexibility. The clustering of false positives in transport genes reveals a systematic gap in transporter substrate specificity representation, and each case constitutes a specific, testable hypothesis for model refinement.

**Fig 8:**
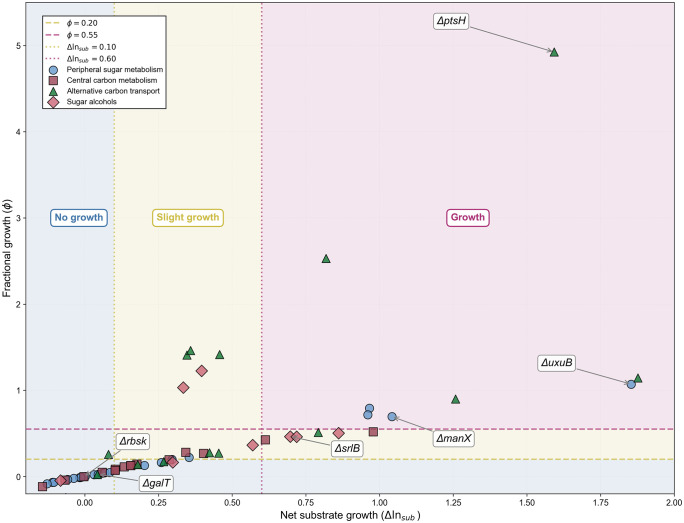
Scatter plot showing the distribution of all tested strains in the two-parameter classification space defined by net substrate growth $(\Delta ln_{sub}$, x-axis) and fractional growth (ϕ, y-axis). Each point represents one strain-substrate combination, with shape and color indicating metabolic category: peripheral sugar metabolism (purple circles), central carbon metabolism (brown squares), alternative carbon transport (teal triangles), and sugar alcohols (magenta diamonds). Background shading indicates classification zones: validated auxotrophs, partial auxotrophs, and false positives. Decision boundaries are shown as dashed lines. Classification requires satisfying both parameter thresholds simultaneously, illustrating the AND logic of the two-parameter system. This representation demonstrates that peripheral sugar metabolism strains (purple circles) cluster predominantly in the validated auxotroph zone, while alternative carbon transport strains (teal triangles) are more dispersed across classification categories, reflecting varying degrees of metabolic redundancy and compensatory pathway availability.

Analysis by metabolic category revealed correlation between pathway specialization and prediction accuracy (Fig 8). Peripheral metabolic pathways achieved 69.2% validation rate, while central carbon metabolism showed only 25.0% suggesting the presence of un-modeled so far metabolic redundancies. Alternative carbon transport and sugar alcohol utilization showed intermediate performance at 66.7% and 62.5%, respectively. The succinate dehydrogenase complex exhibited divergent subunit essentiality results, with deletion strains Δ*sdhC* and Δs*dhD* creating validated auxotrophies (grown on Succinic Acid) whereas Δ*sdhA* and Δ*sdhB* permitted partial growth. Similarly, the Sorbitol operon displayed phenotypic heterogeneity: Δ*srlD* produced validated auxotrophy (ϕ = 0.165), whereas Δ*srlA*, Δ*srlB*, and Δ*srlE* maintained partial growth (ϕ = 0.365−0.464) on d-Sorbitol.

The successful validation of 36 auxotrophic strains (59.0% of tested designs) establishes a roadmap for functional genomics testing. These validated auxotrophs represent strains where computational predictions accurately identified metabolic vulnerabilities suitable for complementation-based gene characterization.

## 4. Discussion

The central contribution of this study is a systematic computational framework for designing conditional auxotrophic strains of *Escherichia coli* as diagnostic phenotypic tools for functional gene discovery. By extending the *i*ML1515 genome-scale metabolic model with 1,643 Boolean regulatory rules, 258 unique auxotrophic strain designs were identified across single, double, and triple gene knockout tiers, collectively mapping to 220 metabolic rescue functions. Each strain grows normally on glucose but fails on a specific alternative carbon source, creating a selective growth condition under which complementation with an uncharacterized gene directly reveals its metabolic function. In this way, the computational framework designs the selective media while the experimental complementation step makes the functional assignment, a distinction that is central to interpreting the scope and utility of the approach.

Experimental validation of 61 single-gene knockout designs from the Keio collection confirmed auxotrophic phenotype in 59% of cases, with a further 28% showing partial auxotrophy. Subsystem-level analysis reveals that prediction accuracy follows a consistent gradient tied to metabolic redundancy. Peripheral metabolic pathways validate at 69.2% and alternative carbon utilization genes at 66.7%, reflecting the fact that peripheral pathway genes typically encode functions with few or no isozymes, such that their deletion creates an insurmountable metabolic block. Central carbon metabolism, by contrast, achieves only 25.0% confirmation, because core metabolic genes are embedded in a highly interconnected network where multiple bypass routes sustain flux despite single-gene loss. False positive predictions are further concentrated in transport genes, particularly PTS system components (*ΔptsH*, *ΔptsI*) and mannose transporters (*ΔmanX*, *ΔmanY*, *ΔmanZ*), which maintained substantial growth across multiple substrates. This likely reflects transporter promiscuity and cross-substrate functionality that is not yet fully represented in the model.

Each category of prediction error points to a distinct refinement target. Transport false positives arise from unmodeled transporter promiscuity and require adding promiscuous substrate uptake routes to the relevant GPR rules and reaction bounds. Central metabolism false positives reflect missing isozyme associations and can be resolved through targeted literature curation of alternative flux-carrying enzymes. Regulatory false positives, where Boolean rules spuriously silence biologically active enzymes, are addressed by correcting the seven reaction rules identified here.

Beyond the specific prediction errors, the Boolean ON/OFF regulatory formulation itself imposes assumptions that bound the accuracy of conditional essentiality predictions. Because regulatory states are treated as binary, the framework cannot capture graded or condition-dependent expression levels, which is particularly consequential at regulatory transitions between carbon sources. Growth-based reactivation, where reactions flagged OFF are restored when the model cannot grow without them, prevents non-biological lethality but may over-permissively activate reactions that should remain silent under specific conditions. Essential gene protection, the rule that prevents reactions indispensable for glucose growth from being silenced by regulatory constraints, similarly introduces a boundary-case risk: genes that are nearly but not unconditionally essential on glucose may be incorrectly held active under alternative substrates, masking conditional essentiality that would otherwise be predicted. As a result, predictions are most reliable for genes governed by well-characterized, strongly switching regulatory circuits and should be interpreted with greater caution for genes in the constitutive default class.

The triple-gene knockout tier explores a new layer of metabolic redundancy that is invisible to single and double knockout screening alone. The seven universally essential gene triples, which are lethal across all 45 non-glucose substrates, reflect pathways where three-way isozyme redundancy has evolved to provide robust metabolic capacity regardless of carbon source. The *aroGHF* DAHP synthase system illustrates this logic directly: three independently regulated isozymes, each feedback-inhibited by a different aromatic amino acid, must all be deleted before aromatic amino acid precursor synthesis is eliminated. Similarly, the *hemH*/*efeB*/*yfeX* ferrochelatase triple reflects a primary plus two secondary porphyrin metalation routes whose combined redundancy sustains heme B synthesis under all growth conditions tested. The high specificity of TKO strains, with 89% carrying exactly one rescue function, arises precisely because three-way deletion of an isozyme family collapses the network to a single obligate route, producing an unambiguous auxotrophic phenotype and a highly selective complementation assay.

The SKO, DKO, and TKO designs serve as a pre-computed filter for complementation library screens, defining which deletion strains to use and what rescue function to expect before experiments begin, consistent with recently demonstrated library screening strategies [44]. Higher-order knockouts beyond triple deletions are computationally feasible but offer diminishing returns in novel rescue functions and increasing experimental complexity.

Extending this framework to non-model organisms would require a complete GSM model, adequate characterization of transport reactions, a regulatory rule set of sufficient scope to correctly identify conditionally essential genes under each substrate, and genetic tools for constructing defined single and multi-gene deletions. Reconstruction gaps inflate false negative rates, transport predictions are the least reliable even in *E. coli*, and unconstrained FBA without regulatory rules significantly overestimates essential genes, limitations that scale proportionally with decreasing curation depth. Organisms approaching *E. coli* across all these dimensions, including *Bacillus subtilis*, *Saccharomyces cerevisiae*, and *Pseudomonas putida*, represent the most tractable near-term targets.

This work establishes a systematic and scalable approach for converting genome-scale metabolic knowledge into experimentally actionable phenotypic assays for gene function. The 258 unique auxotrophic strain designs and 220 mapped rescue functions form a functional genomics resource: the computational framework has been built and 59% of single-gene designs have been experimentally confirmed as genuine auxotrophs, establishing the phenotypic foundation required for downstream complementation. Once complementation experiments are performed, each positive result would directly assign a metabolic function to an uncharacterized ORF, while each negative result would constrain the search space and refine the model. As GSM model quality improves, regulatory rule sets expand, and higher-order knockout designs become experimentally accessible, the framework naturally scales to cover a broader functional space, positioning computational auxotroph design as an iterative, data-driven platform for closing the persistent gap between genomic sequence and functional annotation.

## Supporting information

S1 Table rFBA growth summary across all evaluated conditions. Wild-type FBA growth, rFBA growth, growth impact, and feasibility for the four baseline rFBA validation conditions and the 46 aerobic carbon substrates used in the auxotroph design pipeline.
(XLSX)

S2 Table Reactions inactivated by regulatory rules. The 12,271 (substrate, reaction) combinations in which the rFBA evaluation turns a reaction OFF, with reaction ID, GPR, the OFF gene(s), wild-type FBA flux, and applied/relaxed flag.
(XLSX)

S3 Table Single-gene knockout designs and rescue functions. Deduplicated list of the 97 unique conditionally essential SKO designs and their rescue reactions from the digital reaction library, with match key (EC, BiGG, or MetaNetX), percent wild-type biomass restored, and number of equivalent rescue variants.
(XLSX)

S4 Table Conditionally essential double-knockout gene pairs. All 3,823 conditionally essential gene pairs across the 46 substrates, including pairs without an identified rescue function (rescuable pairs are in S5 Table).
(XLSX)

S5 Table Double-gene knockout designs and rescue functions. Deduplicated list of the 142 unique rescuable DKO designs covering 4,203 (substrate, gene-pair, rescue) combinations from the digital reaction library.
(XLSX)

S6 Table Conditionally essential triple-knockout gene combinations. All 375 irreducible three-way synthetic-lethal gene triples across 45 substrates, filtered as described in Methods.
(XLSX)

S7 Table Triple-gene knockout designs and rescue functions. The 19 unique rescuable TKO designs and their 374 (substrate, triple, rescue) combinations; 373 of 374 records achieve 100 percent wild-type biomass restoration.
(XLSX)

S8 Table Experimental growth phenotypes and threshold sensitivity analysis. Workbook for the 61 tested SKO strains, with sheets for per-strain phenotype summary (raw and water-corrected OD metrics, fractional growth, classification), the 27-threshold sensitivity grid, the 10 borderline strains, the threshold legend, and the analysis settings.
(XLSX)

S9 Table Time-resolved OD_600_ growth curves. Long-format table of replicate mean and standard deviation OD_600_ across the 24 h microplate assay for the 61 tested strains, under substrate, glucose, and water conditions, at 49 time points (8,918 rows).
(XLSX)

S1 Fig Growth curves for the 61 tested auxotrophic single-knockout strains. Multi-panel plot of mean OD_600_ with replicate standard deviation over the 24 h M9 microplate assay, comparing growth on the predicted auxotrophic substrate, glucose (positive control), and a no-carbon control (negative control). Panel labels indicate the deleted gene and the auxotrophic substrate.
(PDF)
