# Supplementary figures and images for: Systematic design of auxotrophic strains and media conditions to probe metabolic functions in E. coli
Source: PLoS Comput Biol. 2026 Jun 29;22(6):e1014469. doi: 10.1371/journal.pcbi.1014469 (PMC13345470; doi:10.1371/journal.pcbi.1014469)

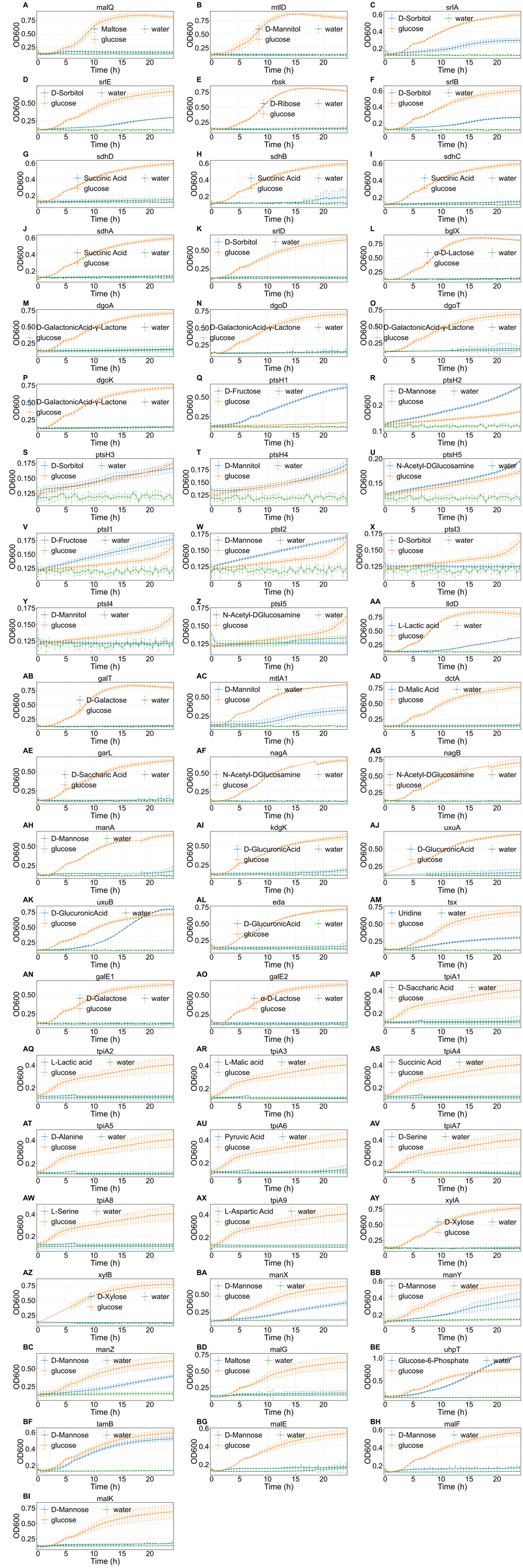

Supplement: S1 Fig — Multi-panel plot of mean OD600 with replicate standard deviation over the 24 h M9 microplate assay, comparing growth on the predicted auxotrophic substrate, glucose (positive control), and a no-carbon control (negative control). Panel labels indicate the deleted gene and the auxotrophic substrate. (PDF) [file pcbi.1014469.s010.pdf]
